# A Primary Pleural Hydatid Cyst in an Unusual Location

**Published:** 2017

**Authors:** Parviz Mardani, Mohammad Yasin Karami, Kamran Jamshidi, Navid Zadebagheri, Hadi Niakan

**Affiliations:** 1 Department of Surgery, Shiraz University of Medical Sciences, Shiraz, Iran.,; 2 Student Research Committee, Faculty of Medicine, Shiraz University of Medical Sciences, Shiraz, Iran.

**Keywords:** Echinococcosis, Pulmonary, Hydatid cyst, Pleura

## Abstract

Hydatid cyst has a predilection to involve the liver and lungs. Most of the reported cases of intra-pleural hydatid cyst are secondary; primary involvement has rarely been reported in the English-language literature. Here, we report on a 33-year-old woman who presented with complaints of dyspnea, cough, low-grade fever, and chills over the previous 3 months. Primary pleural hydatidosis was suspected on abdominopelvic CT; hence, right thoracotomy and cystectomy were performed. Albendazole was administered postoperatively for 6 months. During this period, liver function tests and abdominal sonography results were normal. Despite its rarity, our case emphasizes that general surgeons should suspect primary hydatidosis of the plural cavity when they detect large cystic masses in patients with mediastinal shifting and radiography findings such as white lung, especially in patients with fever and dyspnea.

## INTRODUCTION

Hydatid disease is a zoonotic disease caused by four different *Echinococcosis* species that is endemic in developing countries (1–3). *Echinococcus granulosus* accounts for 95% of the human hydatid cases reported (3).

Carnivores are the definitive hosts and herbivores are the intermediary hosts of the parasite. Humans themselves have no role in the biological life cycle and are usually infected after inadvertent ingestion of *Echinococcosis* eggs in canine feces (4, 5).

Hydatid cysts are usually located in the liver and lung. The rates of localization of hydatid disease in different body organs vary in the literature (1). Intrathoracic, extra pulmonary hydatid cysts are very rare and their features and management require discussion. This case report presents a primary intrapleural hydatid cyst.

## CASE SUMMARIES

**Figure 1.**
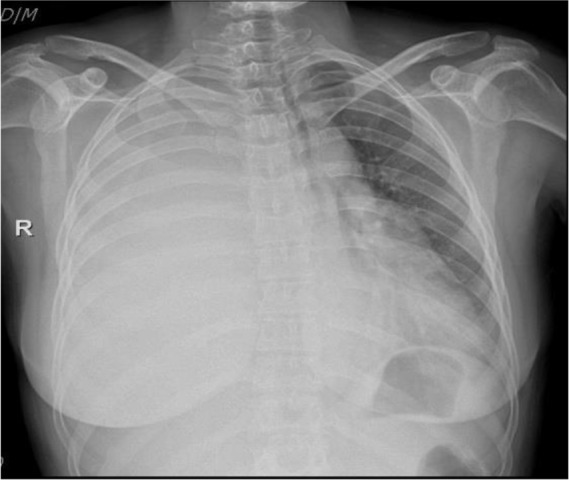
Chest X-Ray radiograph shows right side white lung and mediastinal shifting to the left

A 33-year-old woman presented with complaints of dyspnea, cough, low-grade fever, and chills over the previous three months. On presentation in general examination, the patient was hemodynamically stable, with a pulse rate of 80 beats/minute, a blood pressure of 120/80 mmHg, and an oral temperature of 37.3 °C. Percussion dullness was noted in the right side of the chest, with normal percussion in the left side. On auscultation, the breathing sounds were found to be decreased in the right side of the chest. Chest X-ray revealed right side white lung (Figure 1).

**Figure 2.**
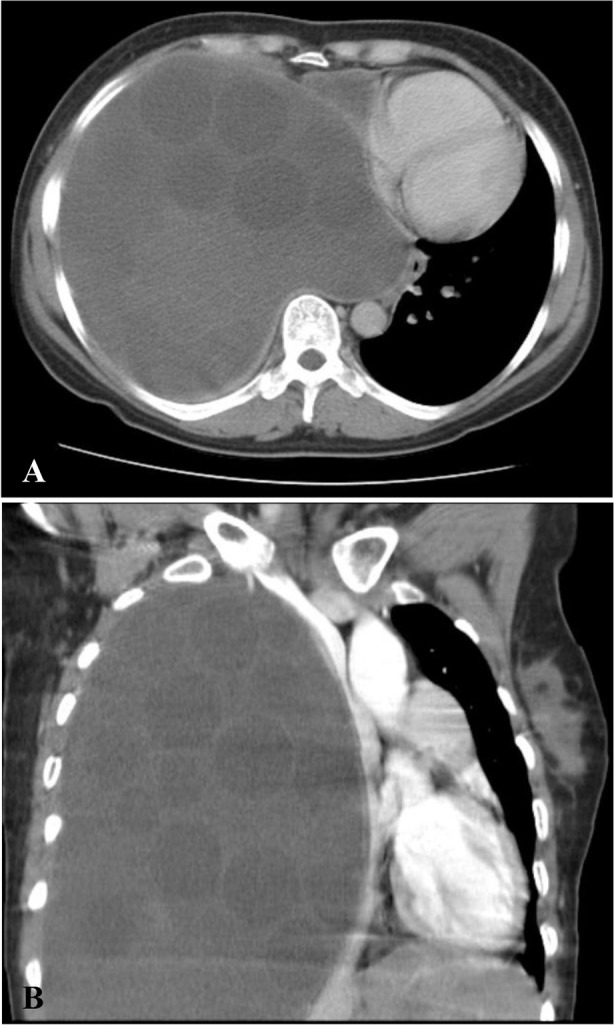
(A) Computed tomography showed multiple cysts filling the entire right pleural area and mediastinal shifting to left. (B) Coronal view computed tomography showed the same finding.

**Figure 3.**
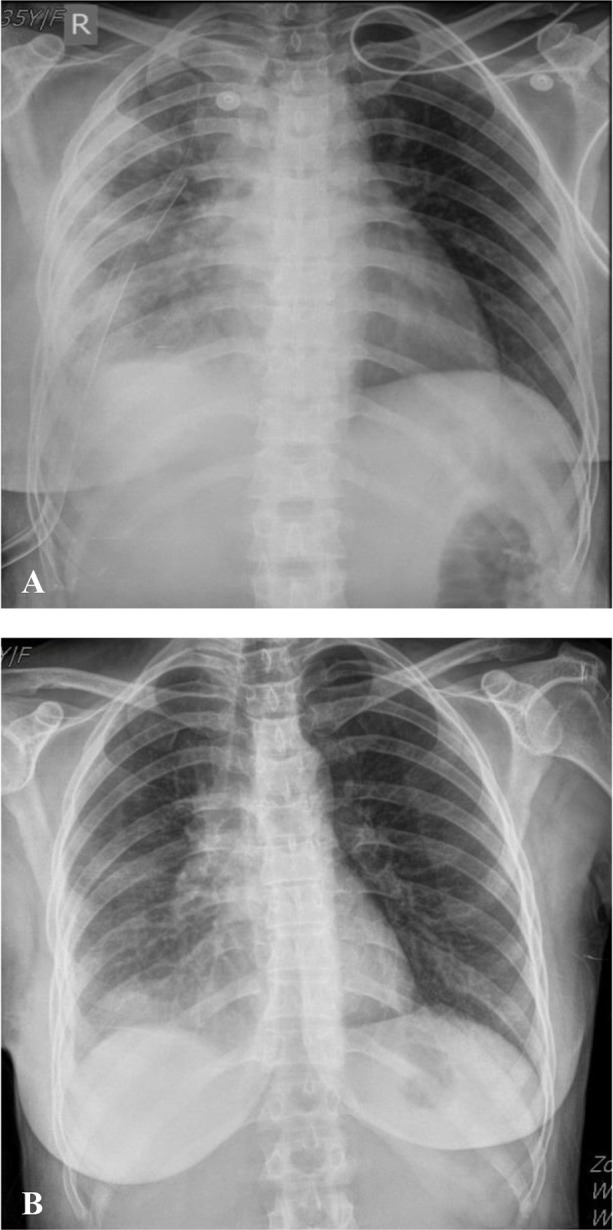
(A) Post-operation chest X-ray radiograph showed expanded right side lungs mild pleural effusion and thoracostomy in it .(B) After one month follow up, chest X-ray radiograph showed a normal pattern.

Computed tomography showed multiple cysts that filled the entire right pleural area (Figure 2). The patient was posted for thoracotomy and right posterior-lateral thoracotomy was performed; the right pleural cavity was entered and the wound was irrigated with hypertonic saline. The large hydatid cyst and its daughter cysts were evacuated completely. The cyst wall was then dissected from the adjacent tissue using both blunt and sharp dissection techniques. The collapsed lung then expanded fully, with no significant parenchymal damage or air leaks. The thoracotomy was closed in layers and the patient was transferred to the intensive care unit (ICU) for elective ventilation. The patient was extubated after 1 day. After 10 days, the patient was discharged from the hospital and had an uneventful recovery. The patient remained asymptomatic in the follow up visits performed one week, one month, and six months post-operatively (Figure 3).

## DISCUSSION

One of the unusual sites for extra-pulmonary hydatid cyst is the intrapleural cavity. Cysts at this site can rupture and release their contents into the pleural cavity, which presents as pneumothorax or hydro pneumothorax (6). The most frequent symptoms are cough, fever, and chest pain (7).

Gursoy et al. (7) reported just one case with extra-pulmonary hydatidosis and pleural involvement. Additionally, Tewari et al. reported a case of primary pleural hydatid cyst with chest wall extension (8). Marghli et al. presented a case of primary heterotopic pleural hydatid cyst presenting as pneumothorax (6). Primary pleural hydatidosis is an extremely rare condition; Erkoç et al. presented a ruptured pleural hydatid cyst in this unusual location (9).

The location of the cyst is a feature of clinical importance, primarily if it is in the pleural layers and the pleural region. In this patient, many cystic structures were drained, and complete decortication of the parietal pleura was performed. No effect on the pulmonary parenchyma was observed.

The pleural layers are avascular, and a hydatid cyst may form and grow in this region because the structure of the laminated cyst membrane is permeable to calcium, potassium, chloride, water, and urea (7). Accordingly, these nutritional substances and others that may be useful to the parasite can traverse the membrane via diffusion (7). Active transport may be involved in this process (10).

In our case, the patient presented with dyspnea, low-grade fever and, as the CT scan showed, the cysts had filled the entire right pleural area, causing mediastinal shifting to the left.

Therefore, complete excision of the large primary intrapleural hydatid cyst was planned to avoid the occurrence of other complications of hydatid cyst, such as rupture, pneumothorax, and compression effects.

## CONCLUSION

Pleural effusion, dyspnea, and cough can be symptoms of an unusual site of extra-pulmonary hydatid disease that can grow and compress the lung, causing symptoms. They are usually suspected upon CT and confirmed intraoperatively. The cyst is carefully dissected from the visceral pleura around the cyst to avoid its rupture into the bronchus and complications such as broncho-pleural fistula and post-operative persistent air leak. To the best of our knowledge, this is an extremely rare case of primary pleural hydatid cyst.
